# Intracellular and Extracellular Redox Status and Free Radical Generation in Primary Immune Cells from Children with Autism

**DOI:** 10.1155/2012/986519

**Published:** 2011-11-24

**Authors:** Shannon Rose, Stepan Melnyk, Timothy A. Trusty, Oleksandra Pavliv, Lisa Seidel, Jingyun Li, Todd Nick, S. Jill James

**Affiliations:** Department of Pediatrics, Arkansas Children's Hospital Research Institute, University of Arkansas for Medical Sciences, Little Rock, AR 72202, USA

## Abstract

The modulation of the redox microenvironment is an important regulator of immune cell activation and proliferation. To investigate immune cell redox status in autism we quantified the intracellular glutathione redox couple (GSH/GSSG) in resting peripheral blood mononuclear cells (PBMCs), activated monocytes and CD4 T cells and the extracellular cysteine/cystine redox couple in the plasma from 43 children with autism and 41 age-matched control children. Resting PBMCs and activated monocytes from children with autism exhibited significantly higher oxidized glutathione (GSSG) and percent oxidized glutathione equivalents and decreased glutathione redox status (GSH/GSSG). In activated CD4 T cells from children with autism, the percent oxidized glutathione equivalents were similarly increased, and GSH and GSH/GSSG were decreased. In the plasma, both glutathione and cysteine redox ratios were decreased in autistic compared to control children. Consistent with decreased intracellular and extracellular redox status, generation of free radicals was significantly elevated in lymphocytes from the autistic children. These data indicate primary immune cells from autistic children have a more oxidized intracellular and extracellular microenvironment and a deficit in glutathione-mediated redox/antioxidant capacity compared to control children. These results suggest that the loss of glutathione redox homeostasis and chronic oxidative stress may contribute to immune dysregulation in autism.

## 1. Introduction

Autism is a behaviorally defined neurodevelopmental disorder that usually presents in early childhood and is characterized by significant impairments in social interaction and communication and by abnormal repetitive hyper-focused behaviors. The prevalence of autism spectrum disorders has increased more than 10-fold in the last two decades, now affecting one in 110 US children, yet the etiology of these disorders remains elusive [1]. Glutathione depletion and oxidative stress have been implicated in the pathology of numerous neurobehavioral disorders including schizophrenia [2], bipolar disorder [3], and Alzheimer's disease [4]. Accumulating evidence suggests that redox imbalance and oxidative stress may also contribute to autism pathophysiology. Multiple biomarkers of oxidative stress have been identified in blood samples from children with autism [5–12]. Our group has reported a decrease in concentrations of glutathione (GSH) and several of its metabolic precursors, an increase in oxidized glutathione disulfide (GSSG), and a decrease in glutathione redox ratio (GSH/GSSG) in case-control evaluations of plasma and lymphoblastoid cell lines derived from children with autism [13–16]. Recently, several interactive polymorphisms in enzymes regulating glutathione synthesis were found to be more prevalent in children with autism suggesting that the glutathione deficit and predisposition to oxidative stress may be genetically based in some children [17]. 

Oxidative stress occurs when cellular antioxidant defense mechanisms fail to counterbalance endogenous ROS production and/or exogenous prooxidant environmental exposures. Glutathione (*γ*-L-glutamyl-L-cysteinylglycine) is a tripeptide that functions as the major intracellular antioxidant and redox buffer against macromolecular oxidative damage. The glutathione thiol/disulfide redox couple (GSH/GSSG) is the predominant mechanism for maintaining the intracellular microenvironment in a highly reduced state that is essential for antioxidant/detoxification capacity, redox enzyme regulation, cell cycle progression, and transcription of antioxidant response elements (ARE) [18–23]. Subtle variation in the relative concentrations of reduced and oxidized glutathione provides a dynamic redox signaling mechanism that regulates these vital cellular processes [24–27]. For example, in both CNS precursor cells and naïve immune cells, intracellular glutathione redox status is the primary determinant modulating the cellular decision to undergo cell cycle arrest, differentiation, or proliferation [27]. A reducing intracellular environment is required for proliferation, while a more oxidized microenvironment favors cell cycle arrest and differentiation. A chronic deficit in the GSH/GSSG redox ratio is considered to be a reliable indicator of oxidative stress and increased vulnerability to oxidative damage from prooxidant environmental exposures [28, 29].

In the extracellular plasma compartment, the cysteine/cystine (thiol/disulfide) redox couple independently provides the ambient redox environment for circulating immune cells. The ambient extracellular cysteine/cystine redox potential has been shown to be more oxidized than the intracellular GSH/GSSG redox potential and is independently regulated [30]. Dynamic shifts in the plasma cysteine/cystine redox potential alter the redox status of cysteine moieties in cell surface proteins to induce conformational changes in protein structure that can reversibly alter function [31, 32]. For example, under oxidizing extracellular conditions, redox-sensitive cysteine residues in the catalytic core of protein tyrosine phosphatases become oxidized and reversibly inactivate enzyme activity depending on the ambient cysteine/cystine redox potential [31, 33, 34]. Extracellular cysteine/cystine redox status is emerging as an important new signal transduction mechanism that can induce posttranslational alterations in downstream redox-sensitive proteins including a variety of enzymes, transcription factors, receptors, adhesion molecules, and membrane signaling proteins resulting in the dynamic modulation of their activity and function [32, 35, 36].

Recent studies have revealed numerous immunologic abnormalities among children with autism including alterations in immune cell proportions [37–40] and shifts in helper T-cell subpopulations after mitogenic stimulation [41, 42]. Peripheral blood mononuclear cells (PBMCs) from individuals with autism have been shown to produce higher levels of proinflammatory cytokines and abnormal levels of regulatory cytokines compared to control PBMCs at baseline and upon mitogenic stimulation [43–46]. Taken together, the immunological studies suggest a role for a dysregulated immune system in autism that potentially could be related to a deficit in glutathione-mediated antioxidant capacity and an oxidized microenvironment in immune cells. To investigate this possibility, we examined whether primary immune cells (PBMCs) from children with autism exhibit decreased intracellular glutathione redox capacity compared to PBMCs from age-matched control children and whether a more oxidized intracellular and extracellular microenvironment is associated with increased production of oxidizing intracellular free radicals. Because immune cells from children with autism have been shown to have abnormal responses to stimulation, we also elected to challenge the PBMCs with immune activators known to promote oxidative stress and measure the resulting intracellular glutathione redox status in activated isolated monocytes and T cells.

## 2. Subjects and Methods

### 2.1. Participants

This investigation was conducted on a subset of children from the autism IMAGE (Integrated Metabolic and Genomic Endeavor) study at Arkansas Children's Hospital Research Institute (ACHRI) that has recruited over 162 case and control families to date. The IMAGE cohort for this study consisted of 43 children diagnosed with autistic disorder and 41 unaffected control children (16 of which were unaffected siblings). The autism case families were recruited locally after referral to the University of Arkansas for Medical Sciences (UAMS), Dennis Developmental Center and diagnosed by trained developmental pediatricians. Children aged 3 to 10 with a diagnosis of autistic disorder as defined by the *Diagnostic and Statistical Manual of Mental Disorders, Fourth Edition* (DSM-IV 299.0), the Autism Diagnostic Observation Schedule (ADOS), and/or the Childhood Autism Rating Scales (CARS >30) were enrolled. Children diagnosed with other conditions on the autism spectrum or rare genetic diseases associated with symptoms of autism were excluded from the study. Children with chronic seizure disorders, recent infection, and high-dose vitamin or mineral supplements exceeding the RDA were also excluded because these conditions are potential confounders that could affect redox status. Unaffected siblings and unrelated, neurotypical children aged 3 to 10 with no medical history of behavioral or neurologic abnormalities by parent report made up the comparison group. The protocol was approved by the Institutional Review Board at UAMS, and all parents signed informed consent.

### 2.2. Materials

Culture flasks, plates, and pipettes were obtained from Corning Life Sciences (Lowell, Mass, USA). RPMI 1640, penicillin/streptomycin, Dulbecco's phosphate-buffered saline (PBS), fetal bovine serum (FBS), and glutamine were purchased from Life Technologies (Carlsbad, Calif, USA). Carboxy-H_2_DCFDA (6-carboxy-2′,7′-dichlorodihydrofluorescein diacetate, diacetoxymethyl ester) was obtained from Molecular Probes (Carlsbad, Calif, USA). Human Monocyte Isolation Kit II and Human CD4 T cell Isolation Kit II were purchased from Miltenyi Biotec (Bergisch-Gladbach, Germany). Histopaque-1077 and all other chemicals were obtained from Sigma-Aldrich (St. Louis, Mo, USA).

### 2.3. Isolation of PBMCs and Stimulation of Monocytes and CD4 T Cells

Fasting blood samples (≤20 mL) were collected before 9:00 AM into EDTA-Vacutainer tubes and immediately chilled on ice before centrifuging at 1300 ×g for 10 min at 4°C. Aliquots of plasma were stored at −80°C in cryostat tubes until extraction and HPLC quantification. PBMCs were isolated by centrifugation over Histopaque-1077. Red blood cells were lysed using a brief (15 s) incubation with 1 mL ice-cold water. Approximately, 30 × 10^6^ PBMCs were resuspended in RPMI 1640 medium (supplemented with 10% FBS, 1% penicillin/streptomycin, and 2 mM glutamine) at a density of 10^6^ cells/mL. Note that because we were unable to obtain 20 mL blood volume from every child, it was not possible to isolate and analyze monocytes and CD4 T cells for all participants. For monocyte stimulation, PBMCs were treated with 0.1 *μ*g/mL lipopolysaccharide (LPS); for T-cell stimulation, PBMCs were treated with 10 ng/mL phorbol 12-myristate 13-acetate (PMA) and 1 *μ*g/mL ionomycin. Cells were placed in a humidified 5% CO_2_ incubator at 37°C for 4 hr. Stimulated monocytes and CD4 T cells were then isolated by negative selection using magnetic cell labeling as described by the manufacturer (Miltenyi Biotec, Bergisch-Gladbach, Germany). Using flow cytometry, we determined that ≥75% of isolated monocytes are positive for CD14 and that ≥87% of isolated CD4 T cells are positive for CD4. For HPLC quantification of GSH and GSSG, approximately 2 × 10^6^ unstimulated (resting) PBMCs, stimulated monocytes, or stimulated CD4 T cells were pelleted, snap frozen on dry ice, and stored at −80°C.

### 2.4. Cell Extraction and HPLC Quantification of Intracellular Glutathione and Plasma Cysteine Redox Status

The storage interval at −80°C before extraction was consistently between 1-2 weeks after blood draw and cell isolation to minimize potential metabolite interconversion. The methodological details for intracellular and extracellular GSH extraction and HPLC elution and electrochemical detection have been described previously [15, 16], and metabolite detection does not require derivatization. Although most GSSG is present as a mixed disulfide with other thiols including cysteine, our measurements detect only the free GSSG in plasma. Glutathione and cysteine concentrations were calculated from peak areas of standard calibration curves using HPLC software. Intracellular results are expressed as nanomoles per milligram of protein using the BCA Protein Assay Kit (Pierce, Rockford, Ill, USA), and plasma results are expressed as micromoles per liter.

### 2.5. Measurement of Intracellular Free Radicals

Carboxy-H_2_DCFDA (DCF) is a membrane-permeable ROS/RNS-sensitive probe that remains nonfluorescent until oxidized by intracellular free radicals. The intensity of DCF fluorescence is directly proportional to the level of free radical oxidation. Approximately, 10^6^ PBMCs were resuspended in 1 mL RPMI 1640 medium supplemented with 10% FBS, 1% penicillin/streptomycin, and 2 mM glutamine and stained in the dark for 20 min with 1 *μ*M DCF at 37°C. Stained cells were washed and resuspended in PBS and analyzed immediately on a Partec CyFlow flow cytometer (Görlitz, Germany) using 488 nm excitation wavelength with 530/30 nm (FL1) emission filter. For each analysis, the fluorescence properties of 10000 cells were collected, and the data were analyzed using the FCS Express software (De Novo Software, Los Angeles, Calif, USA). Intracellular free radical levels are expressed as median fluorescence intensity (MFI) of subject sample DCF fluorescence normalized to DCF fluorescence of a standard PBMC preparation. As an internal control, the standard PBMC preparation was isolated from a 100 mL blood sample from an unaffected healthy adult volunteer, aliquoted and frozen at −180°C in 90% FBS/10% DMSO. An aliquot of the standard PBMC preparation was stained and analyzed with each subject sample. Evaluation of oxidizing free radical production was possible only in those case and unrelated control samples for which sufficient (*∼*20 mL) blood volume was obtained.

### 2.6. Statistical Analysis

Within the control group, 16 of the 41 unaffected control children were case siblings. There were 27 additional case children without a sibling and 25 additional unrelated control children comprising the total case-control cohort of 84 children. To down-weight the impact of outliers, three metabolites observations were curtailed at the extremes of the distributions for PBMC GSH, PBMC GSSG, and Monocytes GSH/GSSG (see footnote in Table 2). The sibling data are correlated resulting in a combined sample of correlated and uncorrelated data; thus, the assumption of all data being independent is not satisfied for the standard two-sample *t*-test. To make use of all data from dependent and independent observations, we used the corrected *Z*-test proposed by Looney and Jones [47]. This statistical approach provides adequate control of Type 1 errors and has more power than a standard Student's *t*-test. Because the DCF data compared cases and unrelated controls (without siblings) the standard Student's *t*-test was used with significance set at 0.05. Nonparametric intercorrelations (Spearman correlation coefficients) between age and gender and the 7 outcome variables, GSH, GSSG, GSH/GSSG, % oxidized glutathione, cysteine, cystine, and cysteine/cystine were determined with the significance level set at 0.05. Data was analyzed using SAS 9.2 software (SAS Institute Inc, Cary, NC, USA). 

## 3. Results

### 3.1. Demographics of Study Population

Table 1 lists the demographics of the study population. The only major differences between cases and controls are that the control group was composed of a greater proportion of females and African Americans, whereas the case group had a greater proportion of Asian subjects. Over-the-counter multivitamin supplement use was higher among cases (39.5%) compared to controls (19.5%); however, the glutathione redox status was statistically unaffected by vitamin use (data not shown).

### 3.2. Decreased Intracellular Glutathione Redox Status in Autism

Table 2 presents the relative intracellular concentrations of GSH, GSSG, the glutathione redox ratio, and the percentage of oxidized glutathione equivalents in resting (unstimulated) PBMCs and in isolated stimulated monocytes and CD4 T cells from children with autism and age-matched control children. The percent oxidized glutathione is expressed in absolute glutathione equivalents as 2GSSG/(GSH+2GSSG). Relative to controls, the intracellular concentration of GSSG and the percent oxidized glutathione were significantly increased (*∼*40%), and the GSH/GSSG ratio decreased (*∼*21%) in PBMCs from children with autism (*P* < 0.001). After stimulation with LPS, monocytes from children with autism also exhibited significantly decreased GSH/GSSG (*∼*31%, *P* = 0.003), increased GSSG concentration (*∼*32%, *P* = 0.01), and 40% higher percent oxidized glutathione (*P* < 0.001). In mitogen-stimulated CD4 T cells from children with autism, the intracellular GSH concentration was *∼*33% lower, the GSH/GSSG was *∼*40% lower (*P* < 0.001), and the percent oxidized glutathione was *∼*55% higher than in stimulated CD4 T cells from control children (<0.001). As expected, activation with LPS and PMA both resulted in decreased intracellular GSH levels and GSH/GSSG in isolated monocytes and CD4 T cells compared to resting (unstimulated) PBMCs. Upon stimulation, there was a greater decrease in intracellular GSH and GSH/GSSG in both CD4 T cells and monocytes from children with autism compared to control children. Neither age nor gender was significantly correlated with any of the outcome measures. The protein content per 10^6^ cells did not differ between case and control children (data not shown).

### 3.3. Decreased Extracellular Glutathione and Cysteine Redox Status in Autism

**Figure 1:**
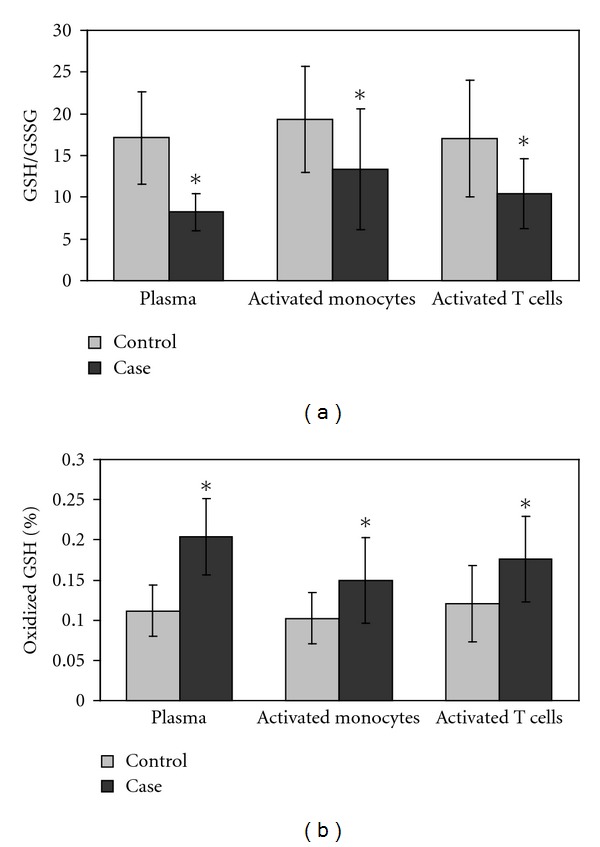
Intracellular and extracellular glutathione redox imbalance in autism. (a) presents the GSH/GSSG in plasma, isolated activated monocytes, and CD4 T cells from case and control children; (b) presents the % oxidized glutathione equivalents. Both extracellular and intracellular glutathione redox status are consistently significantly decreased among the case children (*P < 0.01).

Table 3 presents the relative concentrations of GSH, GSSG, GSH/GSSG, % oxidized GSH, cysteine, cystine, and the cysteine/cystine redox ratio in the extracellular plasma compartment. Children with autism exhibited a significantly decreased extracellular concentration of GSH (*∼*21%) and GSH/GSSG (*∼*54%) and increased concentration of GSSG and the percent oxidized glutathione (52% and 82%, resp., *P* < 0.001). Figures 1(a) and 1(b) compare GSH/GSSG and % oxidized glutathione equivalents, respectively, in plasma, T cells, and monocytes from case and control children and graphically demonstrates the consistent decrease in both extracellular and intracellular glutathione redox status among the case children.

The dynamic balance between the reduced and oxidized forms of glutathione can also be expressed as the redox potential or reducing power of the GSH/GSSG redox couple (*E*_*h*_) and can be calculated from the Nernst equation, *E*_*h*_ = *E*_0_ + *RT*/*nF*ln⁡⁡[disulfide]/([thiol 1]∗[thiol 2]), where *E*_0_ is the standard potential for the glutathione redox couple (−264 mV), *R* is the gas constant (8.314 J/°Kmol), *T* is the absolute temperature of analytical measurement (25°C = 298°K), *n* is 2 for the number of electrons transferred, and *F* is Faraday's constant (96,485 coulomb/mol) [48]. The calculated *E*_*h*_ value for the GSH pool in the children with autism is −116 mV, which is 12 mV more oxidized than in the control children, with an *E*_*h*_ value of −128 mV (Table 3).

The concentration of cystine, the oxidized form of cysteine, was significantly elevated (*∼*52%), while the cysteine/cystine redox ratio was significantly decreased (*∼*31%) in plasma from children with autism (*P* < 0.001). The *E*_*h*_ value for the cysteine pool can also be calculated from the Nernst equation (see above) where the *E*_0_ for cysteine is equal to −250 mV [30]. The calculated *E*_*h*_ value for the cysteine pool in children with autism is −106 mV, or 5 mV more oxidized than the control *E*_*h*_ value of −111 mV (Table 3).

### 3.4. Elevated Free Radical Production in Autism

**Figure 2:**
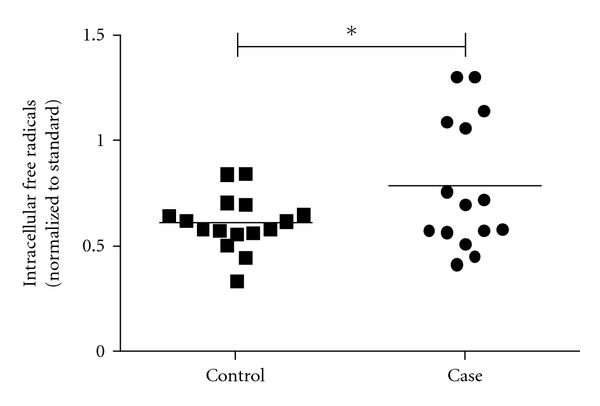
Intracellular Free Radicals are Elevated in Lymphocytes from Children with Autism. Intracellular free radicals were measured in freshly isolated PBMC from children with autism and unaffected control children using 1 uM DCF. Presented is median fluorescent intensity (MFI) of the gated lymphocyte population from subject samples normalized to MFI of a standard PBMC preparation also treated with 1 uM DCF and analyzed with each subject sample. Lymphocytes from children with autism exhibited a significantly higher mean level of intracellular free radicals than controls (*P* = 0.04). Control median (95% CI) = 0.576 (0.551–0.640); case median (95% CI) = 0.689 (0.561–1.086).

The level of intracellular free radicals was measured in available resting PBMCs from children with autism (*n* = 15) and unaffected control children (*n* = 16) using DCF, an ROS/RNS-sensitive fluorescent probe. Monocytes and lymphocytes were gated based on light scatter properties (size and density) and analyzed separately.  Figure 2 presents the median fluorescence intensity (MFI) of lymphocytes from children with autism and unaffected control children (normalized to MFI of the standard PBMC preparation). Gated lymphocytes from children with autism exhibited a significantly higher mean level of intracellular free radicals compared to lymphocytes from control children (*P* < 0.05). No differences in free radical production were observed in gated monocytes from case and control children. Intracellular free radical production was not correlated with age or gender in this cohort.

## 4. Discussion

Oxidative stress is generally defined as an imbalance between oxidant production and endogenous antioxidant defense mechanisms and can be clinically defined in humans by a decrease in the redox status of GSH/GSSG and cysteine/cystine thiol/disulfide redox couples [49]. The relative equilibrium between reduced and oxidized sulfhydryl groups defines the ambient redox state. Low glutathione redox status has been associated with the pathophysiology of several neurobehavioral disorders including schizophrenia [2, 50], bipolar disorder [3], alcoholism [51], HIV [52], and Alzheimer's disease [53]. This is the first study to evaluate intracellular glutathione-mediated antioxidant/redox capacity in primary cells from children with autism as well as the extracellular plasma cysteine/cystine redox status. Because these two redox systems are compartmentalized and independently regulated, evaluation of both redox couples provides a complete picture of the primary immune cell microenvironment in children with autism. Supporting and extending our previous findings of decreased plasma and lymphoblastoid cell GSH/GSSG, we now report that both primary immune cell GSH/GSSG and plasma cysteine/cystine redox couples are similarly compromised resulting in a more oxidized immune cell microenvironment in children with autism compared to control children.

Recent evidence supports the notion that subtle fluctuations in ambient redox status may provide an important regulatory mechanism that can dynamically modulate immune cell function. Activation and proliferation of T cells require a reducing intracellular microenvironment, whereas a more oxidized environment promotes cell cycle arrest and blunted responsiveness to immune stimulation [54–57]. For example, a mechanism involving extracellular redox modulation by regulatory T cells (Tregs) was recently elucidated by Yan et al. [35]. Tregs were shown to inhibit the release of cysteine into the immune synapse between dendritic cells and naïve T cells, which effectively reduces GSH levels in T cells by eliminating the rate-limiting amino acid for GSH synthesis. A high ratio of reduced to oxidized glutathione is required for cell cycle progression from G1 to S phase and induction of the T-cell proliferative response [55]. Thus, the more oxidized GSH/GSSG redox state of the intracellular glutathione pool in PBMCs and in activated CD4 T cells observed in children with autism (Table 2) would suggest a hyporesponsive phenotype that is less conducive to T-cell activation and proliferation. Consistent with this hypothesis, several recent studies have documented abnormalities in the adaptive immune response in children with autism [44, 58].

A glutathione deficit in T cells has been shown to negatively affect the adaptive immune response and T-cell proliferation by reducing IL-2 receptor turnover and IL-2-dependent DNA synthesis [59, 60]. In monocytes, an oxidized intracellular environment has been shown to alter the cytokine profile and skew the Th1 and Th2 balance [61, 62]. Studies in mice have demonstrated that the intracellular GSH content of antigen presenting cells (APCs) reversibly alters the Th1 and Th2 cytokine response pattern [61]. Specifically, a GSH deficit reduced Th1-associated IFN-*γ* production and exaggerated Th2-associated IL-4 production. Restoration of GSH restored the Th1 cytokine response and normalized the Th2 response. Consistent with these observations, two independent studies have reported that helper T-cell subpopulations in PBMCs from children with autism are shifted towards T helper 2 (Th2) dominance [41, 42]. Further, a decrease in T-cell IL-2 receptor expression has been reported to be associated with decreased proliferative response after mitogen stimulation in children with autism [58].

The more oxidized GSH/GSSG redox status in plasma and primary immune cells in children with autism (Figure 1) may offer a mechanistic explanation for the abnormal adaptive immune response previously reported in these children. When intracellular oxidative stress exceeds glutathione redox capacity, cells export GSSG into the plasma as a mechanism to restore internal redox homeostasis [49, 63]. The elevated GSSG concentrations in PBMCs (Table 2) suggest that the GSSG export mechanism and intracellular antioxidant capacity were not sufficient to maintain intracellular redox homeostasis and that redox imbalance was chronic in these children. The association between a more oxidized immune cell microenvironment and an abnormal adaptive immune response warrants continued investigation especially in light of the potential reversibility of immune dysfunction with targeted treatment to restore redox homeostasis [15].

The calculated *E*_*h*_ values for the extracellular GSH and cysteine pools (Table 3) in our control population differ somewhat from previously published values. In adults, the plasma glutathione *E*_*h*_ is more reduced at around −137 mV, and the plasma cysteine redox couple is more oxidized at −80 mV [30, 48]. These discrepancies may reflect methodological differences in sample preparation in that our electrochemical detection does not require derivatization for detection. It is also possible that children (age 3–10 years) may have less reducing capacity than previously reported in adults (age 25–35 years) [48]. Nonetheless, our calculated *E*_*h*_ values are consistent with previous reports that plasma cysteine *E*_*h*_ (−111 mV) is more oxidized than that of GSH (−128 mv).

Mean intracellular free radical production was higher in primary lymphocytes from children with autism relative to lymphocytes from age-matched control children (Figure 2) and was driven by a subset of 5 (33.3%) children whose lymphocytes exhibited especially high levels of free radicals. Mitochondria are the primary producers and targets of intracellular free radicals, and mitochondrial dysfunction has been postulated to be a contributing factor in the pathogenesis of autism and numerous other neurological disorders [64–67]. In a lymphoblastoid cell model, we previously demonstrated that the GSH/GSSG redox ratio in mitochondria was significantly lower in autism compared to control cells and was associated with a significantly lower mitochondrial membrane potential after nitrosative stress [16]. It is well established that mitochondria are highly concentrated in presynaptic terminals and that loss of redox control can negatively affect the efficiency of neurotransmission and synaptic plasticity [68, 69]. Similarly, mitochondrial localization and redox signaling at the immunological synapse between lymphocytes and antigen presenting cells are required for immune activation, and excessive ROS can interrupt these signaling pathways [70–72]. A recent study of mitochondrial defects in lymphocytes from children with autism found decreased complex I activity and overreplication of and deletions in mitochondrial DNA compared to control lymphocytes [73]. Based on this evidence, it is plausible to hypothesize that mitochondria may be the source of the increased levels of lymphocyte free radicals observed in the subset of autistic children presented in Figure 2. Consistent with this hypothesis, a recent meta-analysis estimated that mitochondria dysfunction may affect up to 30% of children with autism [64]. Based on this evidence, further study of mitochondrial function and redox status in lymphocytes from children with autism is warranted.

Relevant to our observations, two recent papers have revealed that an oxidized extracellular cysteine/cystine redox status can initiate a redox signaling cascade that stimulates intracellular mitochondrial ROS production as a mechanism to initiate an inflammatory immune response [74, 75]. The signal transduction from the extracellular to intracellular compartments occurs through oxidative modification of redox-reactive cysteines on cell surface proteins. Exposed cysteine sulfhydryl groups on proteins can be reversibly oxidized to sulfenic acid or disulfide bonds resulting in altered protein structure and function that initiate downstream redox signaling cascades [33, 76, 77]. In an elegant series of experiments, Imhoff and Hansen demonstrated that mitochondrial ROS production was significantly increased in cells incubated under extracellular oxidized cysteine/cystine redox conditions [74]. The stimulated intracellular ROS production resulted in the expression of Nrf-2, the transcription factor responsible for initiation of the inflammatory response. Treatment to block the availability of cell surface cysteine thiol groups abrogated mitochondrial ROS production and Nrf-2 expression. Go et al. confirmed and extended these observations by demonstrating that treatment to maintain mitochondrial redox status abrogated ROS production in the presence of oxidized extracellular cysteine/cystine [75]. Although the precise mechanism for the oxidative cysteine/cystine-dependent signaling for mitochondrial ROS production is not yet clear; the authors provide evidence of a possible link to changes in the redox state of cytoskeletal proteins that could be functionally linked to the mitochondrial membrane. Other studies have demonstrated that an oxidized plasma cysteine/cystine redox potential is associated with proinflammatory conditions [78, 79] and can be modulated by diet [80, 81]. These observations support the possibility that the oxidized plasma cysteine/cystine in children with autism may be functionally related to the increase in lymphocyte free radical production observed and contribute to immune cell abnormalities in these children.

In summary, we show for the first time that both the extracellular and intracellular immune cell compartments are more oxidized in children with autism compared to age-matched unaffected control children. Randomized clinical trials will be needed to determine whether treatment to normalize plasma and intracellular redox status will improve immune cell function and possibly the health and behavior in children with autism.

## Figures and Tables

**Table 1:** Demographics of study population.

	Case children *n* = 43	Control children *n* = 41
Age; mean (SD)	5.42 (1.98)	6.16 (2.29)
Male; *n* (%)	36 (84)	20 (49)
White; *n* (%)	38 (88.4)	31 (75.6)
Asian; *n* (%)	2 (4.65)	0 (0)
African American; *n* (%)	2 (4.65)	8 (19.5)
Hispanic; *n* (%)	1 (2.3)	2 (4.9)
OTC multivitamin use; *n* (%)	17 (39.5)	8 (19.5)

**Table 2:** Intracellular glutathione redox status in resting PBMCs and activated monocytes and CD4 T cells.

Metabolite	Case children		Control children		Corrected *Z*-test	
	*n*	Mean ± SD	*n*	Mean ± SD	Difference (95% CI)	*P* value
Resting PBMCs						
GSH (nmol/mg protein)	43	25.45 ± 8.16	41	23.35 ± 6.38	2.09 (−1.09, 5.29)	0.19
GSSG (nmol/mg protein)	43	0.90 ± 0.3	41	0.66 ± 0.23	0.24 (0.13, 0.35)	<0.001
GSH/GSSG	43	29.58 ± 9.04	41	37.58 ± 10.89	−7.99 (−12.51, −3.48)	<0.001
Oxidized GSH (%)	43	0.07 ± 0.02	41	0.05 ± 0.01	0.02 (0.0075, 0.024)	<0.001

Activated monocytes						
GSH (nmol/mg protein)	18	7.73 ± 3.16	20	8.55 ± 2.5	−0.82 (−2.02, 0.38)	0.18
GSSG (nmol/mg protein)	18	0.62 ± 0.24	20	0.47 ± 0.17	0.14 (0.03, 0.25)	0.01
GSH/GSSG	18	13.31 ± 7.26	20	19.30 ± 6.35	−5.98 (−9.99, −1.97)	0.003
Oxidized GSH (%)	18	0.14 ± 0.05	20	0.10 ± 0.03	0.04 (0.02, 0.07)	<0.001

Activated CD4 T cells						
GSH (nmol/mg protein)	18	6.82 ± 3.0	19	10.16 ± 3.74	−3.33 (−5.24, −1.42)	<0.001
GSSG (nmol/mg protein)	18	0.68 ± 0.29	19	0.63 ± 0.24	0.05 (−0.11, 0.22)	0.51
GSH/GSSG	18	10.47 ± 4.19	19	17.49 ± 6.95	−7.02 (−10.17, −3.87)	<0.001
Oxidized GSH (%)	18	0.17 ± 0.05	19	0.11 ± 0.05	0.05 (0.03, 0.08)	<0.001

GSH: glutathione; GSSG: oxidized glutathione disulfide; oxidized GSH: (%)2GSSG/(GSH+2GSSG); curtailment: PBMC GSH >45 set = 45 (*n* = 1); PBMC GSSG >1.75 set = 1.75 (*n* = 1); Monocytes GSH/GSSG >35 set = 35 (*n* = 1).

**Table 3:** Extracellular (plasma) glutathione and cysteine redox status.

Metabolite	Case children		Control children		Corrected *Z*-test	
	*n*	Mean ± SD	*n*	Mean ± SD	Difference (95% CI)	*P* value
Plasma						
GSH (*μ*M)	38	1.58 ± 0.23	41	1.99 ± 0.22	−0.41 (−0.50, −0.31)	<0.001
GSSG (*μ*M)	38	0.20 ± 0.06	41	0.13 ± 0.04	0.07 (0.05, 0.09)	<0.001
GSH/GSSG	38	8.24 ± 2.20	41	17.14 ± 5.54	−8.73 (−10.52, −6.94)	<0.001
Oxidized GSH (%)	38	0.20 ± 0.05	41	0.11 ± 0.03	0.09 (0.07, 0.10)	<0.001
*E*_*h*_ for GSH		−116 mV		−128 mV		
Cysteine (*μ*M)	41	21.7 ± 4.88	41	21.43 ± 4.08	0.13 (−1.88, 2.14)	0.90
Cystine (*μ*M)	41	29.2 ± 10.6	41	19.26 ± 4.8	9.73 (6.25, 13.2)	<0.001
Cysteine/Cystine	41	0.79 ± 0.18	41	1.14 ± 0.18	−0.33 (−0.41, −0.26)	<0.001
*E*_*h*_ for Cysteine		−106 mV		−111 mV		

GSH: glutathione; GSSG: oxidized glutathione disulfide; *E*_*h*_: steady-state redox potential; *E*_*h*_ for GSH: −264 mV +(30 mV)∗log⁡⁡([GSSG]/[GSH]^2^); *E*_*h*_ for cysteine: −250 mV + (30 mV)∗log⁡⁡([CySSCy]/[Cys]^2^).
